# [Retracted] lncRNA MNX1‑AS1 promotes prostate cancer progression through regulating miR‑2113/MDM2 axis

**DOI:** 10.3892/mmr.2025.13480

**Published:** 2025-03-04

**Authors:** Dong Liang, Chuanjie Tian, Xiaowen Zhang

Mol Med Rep 26: 231, 2022; DOI: 10.3892/mmr.2022.12747

Following the publication of this paper, it was drawn to the Editor's attention by a concerned reader that certain of the cell migration assay data shown in Fig. 2D on p. 5 were strikingly similar to data that had already been published in different form in the journal *OncoTargets and Therapy* in an article written by different authors at different research institutes (which has subsequently been retracted). In addition, there were instances of overlapping data panels identified comparing the data in Fig. 2E and F with those in Fig. 5E and F, such that data which were intended to show the results from differently performed experiments had apparently been derived from the same original sources.

Owing to the fact that the contentious data in the above article had already been published prior to its submission to *Molecular Medicine Reports*, the Editor has decided that this paper should be retracted from the Journal. The authors were asked for an explanation to account for these concerns, but the Editorial Office did not receive a reply. The Editor apologizes to the readership for any inconvenience caused.

